# Time to strategy failure and treatment beyond progression in pretreated metastatic renal cell carcinoma patients receiving nivolumab: *post-hoc* analysis of the Meet-URO 15 study

**DOI:** 10.3389/fonc.2024.1307635

**Published:** 2024-02-12

**Authors:** Veronica Murianni, Alessio Signori, Sebastiano Buti, Sara Elena Rebuzzi, Davide Bimbatti, Ugo De Giorgi, Silvia Chiellino, Luca Galli, Paolo Andrea Zucali, Cristina Masini, Emanuele Naglieri, Giuseppe Procopio, Michele Milella, Lucia Fratino, Cinzia Baldessari, Riccardo Ricotta, Veronica Mollica, Mariella Sorarù, Marianna Tudini, Veronica Prati, Andrea Malgeri, Francesco Atzori, Marilena Di Napoli, Orazio Caffo, Massimiliano Spada, Franco Morelli, Giuseppe Prati, Franco Nolè, Francesca Vignani, Alessia Cavo, Helga Lipari, Giandomenico Roviello, Fabio Catalano, Alessandra Damassi, Malvina Cremante, Pasquale Rescigno, Giuseppe Fornarini, Giuseppe Luigi Banna

**Affiliations:** ^1^ Medical Oncology Unit 1, IRCCS Ospedale Policlinico San Martino, Genoa, Italy; ^2^ Department of Health Sciences (DISSAL), Section of Biostatistics, University of Genoa, Genoa, Italy; ^3^ Medical Oncology Unit, University Hospital of Parma, Parma, Italy; ^4^ Department of Medicine and Surgery, University of Parma, Parma, Italy; ^5^ Medical Oncology Unit, Ospedale San Paolo, Savona, Italy; ^6^ Department of Internal Medicine and Medical Specialties (Di.M.I.), University of Genoa, Genoa, Italy; ^7^ Oncologia 1, Istituto Oncologico Veneto, IOV - IRCCS, Padova, Italy; ^8^ Medical Oncology Department, IRCCS Istituto Romagnolo per lo Studio dei Tumori (IRST) “Dino Amadori”, Meldola, Italy; ^9^ Medical Oncology, Fondazione IRCCS Policlinico San Matteo, Pavia, Italy; ^10^ Medical Oncology 2, Azienda Ospedaliera Universitaria Pisana, Pisa, Italy; ^11^ Department of Oncology, IRCCS, Humanitas Clinical and Research Center, Department of Biochemical Sciences, Humanitas University, Milano, Italy; ^12^ Medical Oncology, AUSL – IRCCS di Reggio Emilia, Reggio Emilia, Italy; ^13^ U.O. Oncologia, IRCCS Istituto Tumori Giovanni Paolo II, Bari, Italy; ^14^ Medical Oncology, Fondazione IRCCS – Istituto Nazionale dei Tumori, Milano, Italy; ^15^ Department of Medical Oncology, Azienda Ospedaliera Universitaria Integrata di Verona, Verona, Italy; ^16^ Department of Medical Oncology, CRO Aviano – Centro di Riferimento Oncologico IRCCS, Aviano, Italy; ^17^ Department of Oncology and Hematology – Oncology Unit, Azienda Ospedaliera Universitaria di Modena, Modena, Italy; ^18^ Oncology Unit, IRCCS MultiMedica, Sesto San Giovanni, Milano, Italy; ^19^ Medical Oncology, IRCCS – Azienda Ospedaliero-Universitaria di Bologna, Bologna, Italy; ^20^ U.O.C. Medical Oncology, Ospedale Camposampiero, Padova, Italy; ^21^ Medical Oncology, Osp. San Salvatore, ASL1 Avezzano Sulmona, L’Aquila, Italy; ^22^ Oncology Unit, Ospedale Michele e Pietro Ferrero, Verduno, Italy; ^23^ Medical Oncology Unit, Policlinico Universitario Campus Bio Medico, Roma, Italy; ^24^ Medical Oncology Department, University Hospital, University of Cagliari, Cagliari, Italy; ^25^ Department of Urology and Gynecology, Istituto Nazionale Tumori IRCCS Fondazione G. Pascale, Napoli, Italy; ^26^ Medical Oncology, Ospedale S. Chiara, Trento, Italy; ^27^ UOC Oncology, Fondazione Istituto San Raffaele Giglio di Cefalù, Cefalù, Italy; ^28^ Medical Oncology Department, Casa Sollievo Della Sofferenza Hospital, IRCCS, San Giovanni Rotondo, Italy; ^29^ Azienda Unità Sanitaria Locale – IRCCS di Reggio Emilia, Reggio Emilia, Italy; ^30^ Medical Oncology Division of Urogenital & Head & Neck Tumors, IEO, European Institute of Oncology IRCCS, Milano, Italy; ^31^ Division of Medical Oncology, Ordine Mauriziano Hospital, Torino, Italy; ^32^ Oncology Unit, Villa Scassi Hospital, Genoa, Italy; ^33^ Medical Oncology, Azienda Ospedaliera per l’Emergenza Cannizzaro, Catania, Italy; ^34^ Department of Health Sciences, Section of Clinical Pharmacology and Oncology, University of Firenze, Firenze, Italy; ^35^ Translationsal and Clinical Research Institute, Centre for Cancer, Newcastle University, Newcastle Upon Tyne, United Kingdom; ^36^ Candiolo Cancer Institute, FPO-IRCCS, Candiolo, Italy; ^37^ Department of Oncology, Portsmouth Hospitals University NHS Trust, Portsmouth, United Kingdom; ^38^ Faculty of Science and Health, School of Pharmacy and Biomedical Sciences, University of Portsmouth, Portsmouth, United Kingdom

**Keywords:** metastatic renal cell carcinoma, immunotherapy, immune checkpoint inhibitors, nivolumab, treatment beyond progression, time to strategy failure, time to treatment failure

## Abstract

**Background:**

Immunotherapies exhibit peculiar cancer response patterns in contrast to chemotherapy and targeted therapy. Some patients experience disease response after initial progression or durable responses after treatment interruption. In clinical practice, immune checkpoint inhibitors may be continued after radiological progression if clinical benefit is observed. As a result, estimating progression-free survival (PFS) based on the first disease progression may not accurately reflect the actual benefit of immunotherapy.

**Methods:**

The Meet-URO 15 study was a multicenter retrospective analysis of 571 pretreated metastatic renal cell carcinoma (mRCC) patients receiving nivolumab. Time to strategy failure (TSF) was defined as the interval from the start of immunotherapy to definitive disease progression or death. This *post-hoc* analysis compared TSF to PFS and assess the response and survival outcomes between patients treatated beyond progression (TBP) and non-TBP. Moreover, we evaluated the prognostic accuracy of the Meet-URO score versus the International Metastatic Renal Cell Carcinoma Database Consortium (IMDC) score based on TSF and PFS.

**Results:**

Overall, 571 mRCC patients were included in the analysis. Median TSF was 8.6 months (95% CI: 7.0 – 10.1), while mPFS was 7.0 months (95% CI: 5.7 – 8.5). TBP patients (N = 93) had significantly longer TSF (16.3 vs 5.5 months; p < 0.001) and overall survival (OS) (34.8 vs 17.9 months; *p* < 0.001) but similar PFS compared to non-TBP patients. In TBP patients, a median delay of 9.6 months (range: 6.7-16.3) from the first to the definitive disease progression was observed, whereas non-TBP patients had overlapped median TSF and PFS (5.5 months). Moreover, TBP patients had a trend toward a higher overall response rate (33.3% vs 24.3%; *p* = 0.075) and disease control rate (61.3% vs 55.5%; *p* = 0.31). Finally, in the whole population the Meet-URO score outperformed the IMDC score in predicting both TSF (c-index: 0.63 vs 0.59) and PFS (0.62 vs 0.59).

**Conclusion:**

We found a 2-month difference between mTSF and mPFS in mRCC patients receiving nivolumab. However, TBP patients had better outcomes, including significantly longer TSF and OS than non-TBP patients. The Meet-URO score is a reliable predictor of TSF and PFS.

## Introduction

The approval of immune-checkpoint inhibitors (ICIs) as monotherapy and immune combinations has deeply changed the prognosis of patients with metastatic renal cell carcinoma (mRCC) (1). Nivolumab (anti-PD1) was the first ICI approved in 2015 for mRCC progressing to anti-angiogenic therapy, based on the results of the CheckMate-025 trial (2). Thereafter, ICI-based combinations became the standard of care for first-line treatment (3) with a reduction in the risk of death up to 35% when compared to sunitinib (4–7).

Patients receiving immunotherapy might develop unique response patterns, including the possibility of tumor burden decrease, durable response or stable disease after initial progression based on conventional response criteria (i.e., Response Evaluation Criteria in Solid Tumors [RECIST] version 1.1) (8). The biological rationale of these atypical patterns of response may be explained by delayed activation of the immune system and apparent tumor burden growth due to transient immune-cell infiltration (9). As a result, in clinical practice ICIs may be continued after radiological progression (treatment beyond progression – TBP) when a clinical benefit is observed (9). For this reason, a modified RECIST V.1.1 for immunebased therapeutics (iRECIST) was developed published in 2017 even though very limited data about its use in prospective clinical trials and real-world clinical experience are available (8).

In this context, progression-free survival (PFS), based on the first disease progression according to RECIST criteria, may not express the real long-term benefit of immunotherapy (10).

The assessment of alternative surrogate endpoints of overall survival (OS) and treatment benefit, like the time to treatment failure (TTF), time to strategy failure (TSF) and time to next treatment (TTNT) has thus become key for clinical practice (11–13).

These clinical-practice reflective endpoints are rarely investigated in clinical trials and emerged as informative endpoints in real-world retrospective analyses of non-small cell lung carcinoma (NSCLC) and melanoma patients receiving ICIs (14–16). However, few analyses described these endpoints in mRCC patients receiving immunotherapy (17, 18).

The Meet-URO 15 study was a multicenter retrospective analysis conducted on 571 mRCC patients receiving nivolumab as ≥ 2^nd^ line treatment, which led to the development of a novel prognostic score, the Meet-URO score, which showed a higher prognostic accuracy compared with IMDC score (available at: https://proviso.shinyapps.io/Meet-URO15_score/) (19).

In this sub-analysis of the study, we aimed to assess the difference between TSF and PFS in the overall study population and the response and survival outcomes between patients treated beyond progression (TBP patients) and those not treated beyond progression (non-TBP patients). In addition, we assessed the prognostic performance of the Meet-URO score versus the IMDC score for both TSF and PFS.

## Methods

Patient characteristics are presented using absolute frequencies and percentages for categorical variables, and medians with ranges for quantitative variables. We evaluated differences in patients’ characteristics between those who had experienced TBP and those who had not, using the standardized mean difference (SMD). An SMD value of less than 0.10 was considered indicative of a well-balanced comparison between the two groups.

Regarding survival and responde outcomes, we considered PFS, TSF, OS, overall response rate (ORR) and disease control rate (DCR).

PFS was defined as the time elapsed from the initiation of therapy to the first instance of radiographic/clinical progression or death, whichever occurred first, with censoring at the last follow-up for patients who were alive without progression.

Conversely, TSF was defined as the time from treatment initiation to the definitive progressive disease which was responsible for the change in the therapeutic line or was not associated with subsequent therapy, or death. This endpoint includes the time elapsed to the first radiological disease progression (as defined by PFS) and next treatment (i.e., TTNT), the discontinuation of immunotherapy for reasons other than progression (i.e., TTF), and the second radiological or clinical disease progression (definitive progression) in those patients who received treatment beyond first radiological disease progression.

To estimate the magnitude of the benefit of TBP, the time (delay) from first to definitive disease progression was also assessed.

The definition of the other endpoints have been already reported in the original Meet-URO paper (19).

Survival estimates were obtained using the Kaplan-Meier method, while univariable and multivariable Cox regression analyses were conducted to evaluate the association between TBP status and both PFS and TSF. In the multivariable analysis, we selected all characteristics that showed an SMD greater than 0.10 between the two groups. Given that the Meet-URO score incorporated the IMDC score, we considered only the Meet-URO score for the multivariable analysis.

The results were reported in terms of hazard ratios (HR) along with their corresponding 95% confidence intervals (95%CI). The statistical significance level was set at 0.05. All statistical analyses were performed using Stata version 16 (StataCorp, 2019).

## Results

### Patients’ characteristics

All 571 patients of the Meet-URO 15 study were included in this analysis (19).

Data on TBP was available in 501 patients (87.7%): 93 patients (18.6%) were TBP patients and 408 patients (81.4%) were non-TBP. Their characteristics are summarized in **Table 1**.

**Table 1:** Patients’ characteristics.

Clinical variables	All population N = 501	TBP pts (N = 93)	Non-TBP pts (N = 408)	SMD^a^
Gender Male Female	359 (71.7) 142 (28.3)	63 (67.7) 30 (32.3)	296 (72.6) 112 (27.5)	0.10
Mean age, years (range) < 70 ≥ 70	63 (52-70) 377/499 (75.6) 122/499 (24.4)	64 (55-71) 68/92 (73.9) 24/92 (26.1)	63 (52-70) 309/407 (75.9) 98/407 (24.1)	0.10
Karnofsky performance status ≥ 80% < 80%	413/496 (83.3) 83/496 (16.7)	78/92 (84.8) 14/92 (15.2)	335/404 (82.9) 69/404 (17.1)	0.05
Histologic subtype Clear cell Non-clear cell	419/497 (84.3) 78/497 (15.7)	76/91 (83.5) 15/91 (16.5)	343/406 (84.5) 63/406 (15.5)	0.082
Nephrectomy Yes No	436 (87.0) 65 (13.0)	81 (87.1) 12 (12.9)	355 (87.0) 53 (13.0)	0.003
Metastatic ad diagnosis Yes No	208 (41.5) 293 (58.5)	40 (43.0) 53 (57.0)	168 (41.2) 240 (58.8)	0.037
Nivolumab line 2^nd^ line 3^rd^ line ≥ 4^th^ line	343 (68.5) 105 (21.0) 53 (10.5)	60 (64.5) 25 (26.9) 8 (8.6)	283 (69.4) 80 (19.6) 45 (11.0)	0.018
IMDC score at start of nivolumab Favourable Intermediate Poor	105/488 (21.5) 319/488 (65.4) 64/488 (13.1)	26/91 (28.5) 60/91 (66.0) 5/91 (5.5)	79/397 (19.9) 259/397 (65.2) 59/397 (14.9)	** ** 0.32
Meet-URO score at start of nivolumab 1 2 3 4 5	67 (13.7) 162 (33.2) 142 (29.1) 91 (18.7) 26 (5.3)	17 (18.7) 36 (39.5) 25 (27.5) 11 (12.1) 2 (2.2)	50 (12.6) 126 (31.7) 117 (29.5) 80 (20.2) 24 (6.0)	0.34
Bone metastases Yes No	187 (37.3) 314 (62.7)	33 (35.5) 60 (64.5)	154 (37.8) 254 (62.2)	0.047

Pts, patients; N number of patients; IMDC, International Metastatic RCC Database Consortium; TBP, treatment beyond progression; SMD, Standardized Mean Difference.

Gender and age were slightly unbalanced between the two groups with a higher percentage of females and over 70 years in the TBP group. Furthermore, the two groups were unbalanced for the IMDC and Meet-URO score subgroups with a lower percentage of patients with poor prognosis in the TBP group (IMDC poor-risk: 5.5% vs 14.9%; Meet-URO score group 4: 12.1% vs 20.2% and group 5: 2.2% vs 6.0%).

### Survival outcomes of overall population

At the time of data cut-off (April 2023), patients were followed up for a median of 21.6 months. The median OS (mOS) was 25.4 months (95% CI: 21.4- 30.5), mPFS 7.0 months (95% CI: 5.7 – 8.5) and mTSF 8.6 months (95% CI: 7.0 – 10.1).

### Survival outcomes of TBP patients

**Figure 1:**
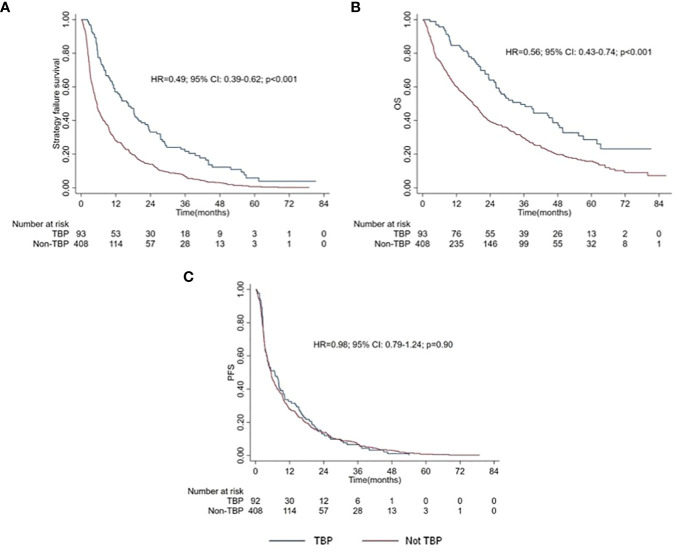
Kaplan Meiers curves of TSF**(A)**, OS **(B)** and PFS **(C)** between TBP patients and Non-TBP patients.

A trend toward a higher ORR (33.3% vs 24.3%; *p* = 0.075) and disease control rate (61.3% vs 55.5%; *p* = 0.31) to nivolumab was observed in TBP compared to non-TBP patients. TBP patients had longer TSF (16.3 vs 5.5 months; *p* < 0.001) (**Figure 1A**) and OS (34.8 vs 17.9 months; *p* < 0.001) (**Figure 1B**), but similar PFS (**Figure 1C**) compared to non-TBP patients (*p* = 0.89) (**Table 2**).

**Table 2:** Response and survival outcomes in TBP and Non-TBP patients.

	TBP pts	Non-TBP pts	*p* value
	(N, %)		
Response outcomes			
ORR (%)	31/93, 33.3%	99/407, 24.3%	0.075
DCR (%)	57/93, 61.3%	226/407, 55.5%	0.31
Response outcomes			
Median PFS (mo) (95% CI)	6.7 (3.8-8.8)	5.5 (4.7-6.2)	0.89
Median TSF (mo) (95% CI)	16.3 (11.2-19.9)	5.5 (4.7-6.2)	**<0.001**
Median delay from first to definitive progression (mo) (95% CI)	4.1 (3.7-6.0)	0	**<0.001**
Median OS (mo) (95% CI)	34.8 (26.1-46.2)	17.9 (15.1-20.4)	**<0.001**

N, number of patients; TBP, treatment beyond progression; pts, patients; ORR, objective response; DCR, disease control rate; PFS, progression free survival; mo, months; TSF, time to strategy failure; OS, overall survival. The bold values indicate statistically significant results for the study.

In TBP patients, a median delay of 9.6 months from first to definitive disease progression was observed (16.3 vs 6.7 months), while non-TBP patients had overlapping TSF and PFS (both 5.5 months).

After multivariable analysis, adjusting for age, gender, MSKCC score, Meet-URO score and both visceral and lymph node metastases, a longer TSF (HR = 0.46; 95% CI: 0.36-0.59; *p* < 0.001) and OS (HR = 0.54; 95% CI: 0.40-0.72; *p* < 0.001) for TBP patients were confirmed (**Table 3**).

**Table 3:** Multivariable analysis of TSF and OS between TBP pts and non-TBP pts.

	TSF	OS
	HR (95% CI); *p* value	
**TBP pts vs non-TBP pts**	0.46 (0.36-0.59); *p* < 0.001	0.54 (0.40-0.72); *p* < 0.001
**Age**	0.99 (0.99-1.00); *p* = 0.088	1.00 (0.99-1.01); *p* = 0.70
**Gender** (Female vs Male)	1.16 (0.95-1.43); *p* = 0.14	1.23 (0.98-1.55); *p* = 0.072
MSKCC score		
1	1.00 (ref)	1.00 (ref)
2	1.40 (0.98-1.99); *p* = 0.064	1.33 (0.88-2.00); *p* = 0.18
3	1.80 (1.10-2.94); *p* = 0.019	1.77 (1.04-3.03); *p* = 0.036
Meet-URO score		
1	1.00 (ref)	1.00 (ref)
2	1.13 (0.77-1.66); *p* = 0.54	1.47 (0.92-2.37); *p* = 0.11
3	1.17 (0.74-1.84); *p* = 0.50	1.83 (1.06-3.14); *p* = 0.029
4	1.54 (0.94-2.51); *p* = 0.086	3.05 (1.73-5.40); *p* < 0.001
5	4.07 (2.22-7.47); *p* < 0.001	6.35 (3.24-12.46); *p* < 0.001
**Lymphnode metastases**	0.93 (0.78-1.12); *p* = 0.47	1.09 (0.89-1.34); *p* = 0.40
**Visceral metastases**	1.01 (0.75-1.36); *p* = 0.94	0.99 (0.72-1.37); *p* = 0.95

Pts, patients; TBP, treatment beyond progression; TSF, time to strategy failure; OS, overall survival; MSKCC, Memorial Sloan–Kettering Cancer Center; HR, hazard ratio; CI, confident interval.

### Survival outcomes and Meet-URO score

**Figure 2:**
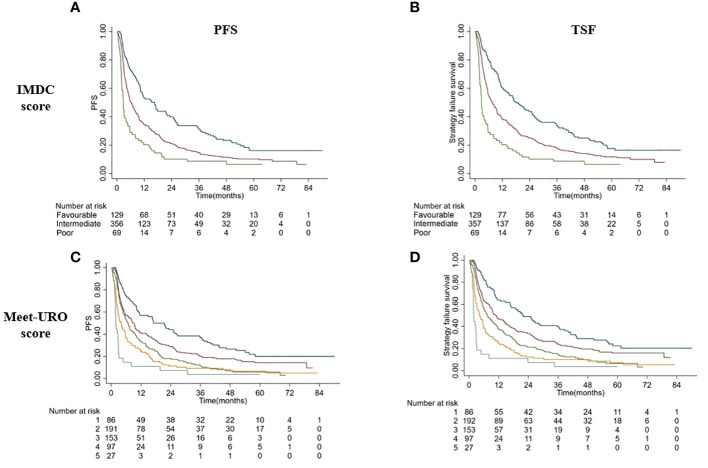
Kaplan Meiers curves of PFS **(A, C)** and TSF **(B, D)** according to IMDC score **(A, B)** and Meet-URO score **(C, D)**.

Applying the Meet-URO score in the overall population according to the two progression outcomes, the Meet-URO score performed better than the IMDC score in both TSF (c index: 0.627 vs 0.594) and PFS (0.616 vs 0.587) (**Figure 2**, **Table 4**).

**Table 4:** Correlation between IMDC and Meet-URO scores according to PFS and TSF.

Prognostic score	Median PFS (mo, range)	C index	Median TSF (mo, range)	C index
*IMDC score*		0.587		0.594
Favorable	16.6 (10.3-21.8)		18.6 (12.6-25.2)	
Intermediate	6.2 (5.3-7.6)		7.6 (5.9-9.5)	
Poor	2.8 (2.1-4.0)		2.8 (2.2-4.0)	
*Meet-URO score*		0.616		0.627
1	17.0 (10.7-26.4)		21.6 (16.6-35.7)	
2	9.2 (5.6-11.1)		10.9 (8.5-14.5)	
3	6.7 (4.9-8.9)		7.5 (5.6-10.3)	
4	4.0 (2.8-5.6)		4.2 (3.0-5.7)	
5	1.9 (1.7-2.8)		1.9 (1.7-2.8)	

PFS, progression-free survival; TSF, Time to strategy failure; mo, months; IMDC, International Metastatic RCC Database Consortium.

## Discussion

The evaluation of tumor responses in the era of immuno-oncology is becoming increasingly important with the rapid expansion of indications and approvals of ICIs. With chemotherapy, efficacy usually correlates with tumor shrinkage as per RECIST criteria (20), while with immunotherapy, clinicians have been facing new patterns of disease response, like pseudo-progression or sustained responses after initial radiological progression. In this context, TBP has become a therapeutic strategy when a clinical benefit is observed (9).

Real-world experiences in the management of NSCLC and melanoma have already reported survival benefits for TBP patients, regardless of the best response to immunotherapy, whether disease control or progression (21, 22). In the retrospective OAK study, 168 NSCLC patients who continued atezolizumab after disease progression, had a longer OS (12.7 vs 8.8 months) than those who switched to other treatments (23). Similarly, the mOS in patients with metastatic squamous cell carcinoma of the head and neck receiving nivolumab beyond progression was 12.7 months vs 8.8 months in the overall population (24).

Regarding mRCC, in the sub-analysis of the Checkmate-025 study, 13% of patients receiving nivolumab beyond progression experienced a subsequent ≥ 30% decrease in tumor burden (25). Tumor burden reduction was observed in patients who initially responded and then progressed, as well as in patients with stable disease or progressive disease as their best response (25). However, even if the study met its primary endpoint of OS, the benefit in PFS over everolimus was not statistically significant. Therefore, classical endpoints such as PFS calculated on the first disease progression might not be a good surrogate endpoint of the real benefit of immunotherapy.

The multicenter retrospective Meet-URO 15 study involved 571 mRCC patients treated with nivolumab as second or beyond treatment line and demonstrated that the prognostic accuracy of the IMDC score, originally developed on patients treated with tyrosine kinase inhibitors, could be improved by the incorporation of neutrophil-to-lymphocyte ratio (NLR) and the presence of bone metastases (e.g., the Meet-URO score) (19)

In this sub-analysis of the Meet-URO 15 study, we evaluated the correlation between TSF and PFS as well as response and survival outcomes in TBP patients and non-TBP patients.

In patients progressing to ≥ 2^nd^ line nivolumab, we observed a relatively small difference of about 2 months between mTSF and mPFS in the overall population. Patients who received nivolumab beyond the first radiological progression had more than doubled TSF although similar median PFS betweenTBP and non-TBP patients, with OS prolonged of 15 months.

Considering TSF as an estimate of immunotherapy benefit, we observed a median delay of more than a year from the first to the definitive disease progression. This paradoxical finding can be attributed to the delayed development of an immune response, which can occur after initial growth of an indicator lesion or the appearance of new lesions. Although RECIST-based PFS has been commonly used as a surrogate endpoint for OS to evaluate novel anti-cancer therapies in clinical trials (26), several ICIs trials have revealed a poor correlation between PFS and OS (10, 27). Although the investigator’s choice to continue treatment beyond the first radiological progression might have been driven by tolerability and favorable clinical features, our findings point out the clinical need for alternative endpoints associated with survival and clinical benefit from ICIs given their unconventional patterns of response.

We have also evaluated the prognostic accuracy of the Meet-URO score in predicting outcomes in this particular setting of patients receiving nivolumab beyond progression. The Meet-URO score incorporates into the IMDC score two independent prognostic factors confirmed with ICIs in mRCC: the presence of bone metastases and an index of inflammation from peripheral blood, namely the NLR (19). The Meet-URO score has demonstrated more accuracy than IMDC in prognostic stratification of pre-treated mRCC patients receiving nivolumab or cabozantinib, likewise nivolumab plus ipilimumab in first-line therapy (28, 29). In this analysis, the Meet-URO score confirmed more accuracy than IMDC for both TSF and PFS.

We acknowledge among study limitations its retrospective nature and the lack of objective assessment of physicians’ preferences leading to treat patients beyond the first radiological progression. Definitions of TBP may differ depending on the scheduled therapy and timing of radiographic assessment. Therefore, our results may be affected by selection bias due to an enrichment in patients with favorable (not measured) clinical features in the TBP group. However, TSF has shown to be a better endpoint of the long-term benefit of immunotherapy and the clinical intuition beyond the imaging alone according to the rigorous RECIST criteria compared with PFS.

Another limitation is that our study involved only patients receiving ICI monotherapy, so the applicability of our results to first-line ICI-TKI combinations and salvage immunotherapy after progression to immunotherapy (e.g. CONTACT-3 trial (30)) is currently limited.

In conclusion, our analysis underscores the potential survival and treatment benefit of immunotherapy beyond progression in pretreated mRCC patients who, in the judgment of their physician, could still derive benefit from nivolumab treatment at the time of the first RECIST disease progression. These findings are consistent with other TBP analyses in solid tumors, such as melanoma, NSCLC and head/neck carcinoma (21–24). Furthermore, the present analysis confirms the higher prognostic accuracy of Meet-URO than IMDC score in stratifying the prognosis of patients with mRCC treated with ICIs. Further prospective investigations are needed to better select patients who may derive benefit from TBP and, in this context, our results will be assessed in the ongoing multicenter prospective study on the first-line therapy of mRCC (Meet-URO 33 – REGAL study) (31).

## Data availability statement

The raw data supporting the conclusions of this article will be made available by the authors, without undue reservation.

## Ethics statement

The studies involving humans were approved by Regional Ethical Committee of Liguria. The studies were conducted in accordance with the local legislation and institutional requirements. The participants provided their written informed consent to participate in this study.

## Author contributions

VMu: Conceptualization, Investigation, Writing – original draft. AS: Conceptualization, Data curation, Formal analysis, Writing – original draft. SB: Conceptualization, Investigation, Writing – review & editing. SR: Conceptualization, Data curation, Investigation, Supervision, Writing – original draft. DB: Investigation, Writing – review & editing. UD: Investigation, Writing – review & editing. SC: Investigation, Writing – review & editing. LG: Investigation, Writing – review & editing. PZ: Investigation, Writing – review & editing. CM: Investigation, Writing – review & editing. EN: Investigation, Writing – review & editing. GPro: Investigation, Writing – review & editing. MM: Investigation, Writing – review & editing. LF: Investigation, Writing – review & editing. CB: Investigation, Writing – review & editing. RR: Investigation, Writing – review & editing. VMo: Investigation, Writing – review & editing. MSo: Investigation, Writing – review & editing. MT: Investigation, Writing – review & editing. VP: Investigation, Writing – review & editing. AM: Investigation, Writing – review & editing. FA: Investigation, Writing – review & editing. MD: Investigation, Writing – review & editing. OC: Investigation, Writing – review & editing. MSp: Investigation, Writing – review & editing. FM: Investigation, Writing – review & editing. GPra: Investigation, Writing – review & editing. FN: Investigation, Writing – review & editing. FV: Investigation, Writing – review & editing. AC: Investigation, Writing – review & editing. HL: Investigation, Writing – review & editing. GR: Investigation, Writing – review & editing. FC: Investigation, Writing – review & editing. AD: Investigation, Writing – review & editing. MC: Investigation, Writing – review & editing. PR: Supervision, Writing – review & editing. GF: Writing – review & editing, Conceptualization, Investigation. GB: Writing – review & editing, Conceptualization, Investigation.
